# Use of mechanical and behavioural methods to eliminate female *Aedes aegypti* and *Aedes albopictus* for sterile insect technique and incompatible insect technique applications

**DOI:** 10.1186/s13071-019-3398-7

**Published:** 2019-03-28

**Authors:** Nayana Gunathilaka, Tharaka Ranathunge, Lahiru Udayanga, Asha Wijegunawardena, Jeremie Roger Lionel Gilles, Wimaladharma Abeyewickreme

**Affiliations:** 1 Department of Parasitology, Faculty of Medicine, University of Kelaniya, Ragama, Sri Lanka; 2 Molecular Medicine Unit, Faculty of Medicine, University of Kelaniya, Ragama, Sri Lanka; 3 Department of Biosystems Engineering, Faculty of Agriculture & Plantation Management, Wayamba University of Sri Lanka, Makadura, Sri Lanka; 4 Insect Pest Control Laboratory, Joint FAO/IAEA Division of Nuclear Techniques in Food and Agriculture, International Atomic Energy Agency, Vienna, Austria; 5 Department of Paraclinical Sciences, Faculty of Medicine, Sir John Kotelawala Defence University, Ratmalana, Sri Lanka

**Keywords:** *Aedes*, Sex separation, Ivermectin, Spinosad, Fay-Morlan glass plate method

## Abstract

**Background:**

Sex separation of mosquitoes at different stages is currently being attempted to ensure the successful release of male mosquitoes in novel vector control approaches. Mechanical and behavioral techniques have been tried most frequently.

**Methods:**

Batches of (*n* = 300) *Aedes aegypti* and *Ae. albopictus* pupae were used for standard sieving (using sieves with 1.12, 1.25, 1.40 and 1.60 mm mesh sizes) and the Fay-Morlan glass plate separation methods. Male and female separation by each method was calculated. For behavioral separation, a spiked blood meal with different concentrations (0, 2, 4, 6, 8 and 10 ppm) of ivermectin and spinosad (spinosyn, 12% w/v), were provided to a batch (*n* = 300) of adult *Ae. aegypti* and *Ae. albopictus* (1:1 sex ratio) followed by observation of mortality. An additional “double feeding method” involved provision of a further blood meal after 24 h, with the same concentrations of ivermectin and spinosad as the initial feeding, followed by a 48-h observation of mortality. All experiments were repeated five times.

**Results:**

In the standard sieving method, the percentage of males and females separated at different pore sizes differed significantly (*P* < 0.05). The majority of the male pupae were collected in the 1.12 mm pore sized sieve for both *Ae. aegypti* (73%) and *Ae. albopictus* (69%) while females were retained mainly in the sieve with the pore size of 1.25 mm. In the Fay-Morlan glass plate separation, 99.0% of the *Ae. aegypti* and 99.2% of the *Ae. albopictus* introduced male pupae could be separated, but with female contaminations of 16 and 12%, respectively. Provision of a blood meal spiked with 8 ppm of ivermectin under the “double feeding” was identified as the most effective way of achieving 100% female elimination for both *Aedes* species.

**Conclusions:**

With 100% separation, use of a spiked blood meal is a more effective method of sex separation than the mechanical methods. Application of the spiked blood meal approach as a second separation level for sexes, after applying the Fay-Morlan glass plate method, could achieve 100% sex separation of sexes whilst allowing a reduction in the amount of toxicants required.

## Background

Mosquitoes are considered the most dangerous vectors of human diseases in the world [1]. *Aedes aegypti* and *Aedes albopictus* (Diptera: Culicidae) are highly anthropophilic mosquitoes [2, 3] and in Sri Lanka have been identified as the primary and secondary vectors of dengue, respectively, which is considered the most important national arboviral disease and a global public health problem [3]. Recently, these vectors have gained a new importance, since they are responsible for the transmission of chikungunya and Zika [4, 5].

In Sri Lanka, clinical dengue-like illness has been recorded since the beginning of this century [6]. At present, the disease comes in epidemic form at 2- to 3-year intervals involving high morbidity [7]. Sri Lanka recently experienced its worst epidemic of dengue, with a total of 186,101 suspected dengue cases in 2017, the highest number ever recorded, while 51,591 suspected dengue cases were reported in 2018 [8].

A major problem is the absence of effective drugs and vaccines for dengue. As a result, the main focus is to reduce the vector densities [9]. In view of the drawbacks associated with conventional mosquito control measures, such as development of insecticide resistance, attempts to develop novel strategies are required for use as part of an integrated vector control strategy [10]. Examples of such novel strategies are the sterile insect technique (SIT) [11] and incompatible insect technique (IIT) [12, 13].

The SIT is a species-specific method that can be used against a range of insect pests and vectors. There is an increasing demand for exploration of the potential applicability of SIT in area-wide integrated vector management (AW-IVM) in many countries [13]. Sterility of male insects can be accomplished with ionizing irradiation and SIT focuses on the release of sufficient sterile males to induce sterility in the wild females which, over time, causes the target population to decline [11]. Through repeated releases of sterile male insects, SIT has the ability to suppress the existing vector populations [14].

Use of endosymbiotic *Wolbachia* is a method to induce sterility in *Aedes* mosquitoes, and is referred to as an incompatible insect technique [15–20]. *Wolbachia* are maternally inherited alphaproteobacteria commonly found in arthropods [21]. These bacteria naturally infect some medically important mosquito species such as *Culex pipiens* [22–24] and *Ae. albopictus* [16]. In addition, *Wolbachia* infections can be performed artificially through microinjection under laboratory conditions. Several mosquito species including *Ae. albopictus* [25–27] and *Ae. aegypti* [28, 29] are currently being infected artificially. In mosquitoes, *Wolbachia* induce a form of embryonic death called cytoplasmic incompatibility (CI) [30, 31] due to sperm-egg incompatibility, when *Wolbachia* infected males mate with either uninfected females or females infected with an incompatible *Wolbachia* strain.

When a mosquito release programme is considered, mass production of mosquitoes is essential. Elimination or separation of females is one of the major issues to be addressed, since females can act as disease vectors by transmitting the infection to humans and mosquito offspring. Therefore, release of females into the environment in such programmes may increase the transmission potential of the target disease. Hence, an efficient sex-separation or female elimination strategy is required for successful implementation of such approaches. Future options may become available using transgenic mosquitoes, but the inherent characteristics of the target species also provide possibilities for separation, at least until more efficient methods can be developed.

Differences in intrinsic size, behavior and development rate between females and males are often present and useful for sexing [13]. The mostly frequently attempted methods broadly include genetic sex separation techniques, molecular methods, and mechanical and behavioral methods. Mechanical and behavioral tools for sexing may be quicker to develop than genetic methods, in which isolation of sex-specific markers may be a challenging, labor-intensive and serendipitous process [13].

For all blood-feeding mosquitoes, sex separation could be achieved at the adult stage *via* behavioral separation by spiking blood with insecticides or other mosquito toxins. Ivermectin is a macrocyclic lactone extract of the *Streptomyces avarnitalis* bacteria. It directly affects the nervous system and muscle function in invertebrates including insects, by inhibiting neurotransmission at glutamate-gated chloride channels. Hence, ivermectin could be used for female elimination in mass-rearing facilities that produce male mosquitoes to be used for IIT and SIT [32].

Spinosad is a naturally-derived insecticide, comprising of a mixture of two neurotoxic macrolide compounds: spinosyn A and D, produced by the fermentation of the Gram-positive soil-dwelling actinomycete *Saccharopolyspora spinosa*. Spinosad acts on the postsynaptic nicotinic acetylcholine and GABA receptors, resulting in tremors, paralysis and death when ingested by the insect [33].

Even though evaluation of sex separation methods is a pre-requisite in both SIT and IIT approaches, no attempt has been made in Sri Lanka to evaluate the efficacy of these methods to be used in the separation of sexes in vector mosquitoes. Therefore, a proper evaluation should be conducted of sex separation of *Aedes* vectors under mass rearing conditions, prior to the application of this technique in the country.

The present study focused on evaluating the efficacy of behavioral (*via* a spiked blood meal treated with ivermectin and spinosad) and mechanical (sieving and Fay-Morlan glass plate separator) sex separation methods in eliminating female *Ae. aegypti* and *Ae. albopictus* mosquitoes under laboratory conditions, with the intention of future use for the SIT and IIT approaches.

## Methods

### Mosquito rearing

The *Ae. aegypti* and *Ae. albopictus* strains used in this experiment originated from eggs collected from the Narangodapaluwa Public Health Inspector (PHI) division, located within the Gampaha District of Sri Lanka in 2015. Both colonies have been kept for four generations at the insectary of the Molecular Medicine Unit, Faculty of Medicine, University of Kelaniya, Sri Lanka, where the experiments were carried out. Adults were maintained under standard laboratory conditions [26 ± 1 °C, 75–80% relative humidity (RH) and a photoperiod of 12:12 h (L:D) in 30 × 30 × 30 cm cages] with 10% sucrose solution provided *ad libitum*. Females were given a blood meal of cattle origin using the Hemotek (PS-6 System, Discovery Workshops, Accrington, UK) artificial membrane feeder.

The larvae were fed with a mixture made of 9.0 g of bovine liver powder, 3.5 g of brewer’s yeast and 12.5 g of tuna meal mixed with 100 ml of distilled water [34]. The larval diet was added once a day in to the tray (25 × 25 × 7 cm^3^), containing 750 L1 larvae in 1000 ml of de-ionized water according to the following regimes: day 1, 1.5 ml (0.50 g/larva); day 2, 1.55 ml (0.52 g/larva); day 3, 1.6 ml (0.53 g/larva); day 4, 1.65 ml (0.55 g/larva); day 5, 1.7 ml (0.57 g/larva).

### Evaluation of mechanical sex separation methods

#### Standard metal sieving

**Fig. 1:**
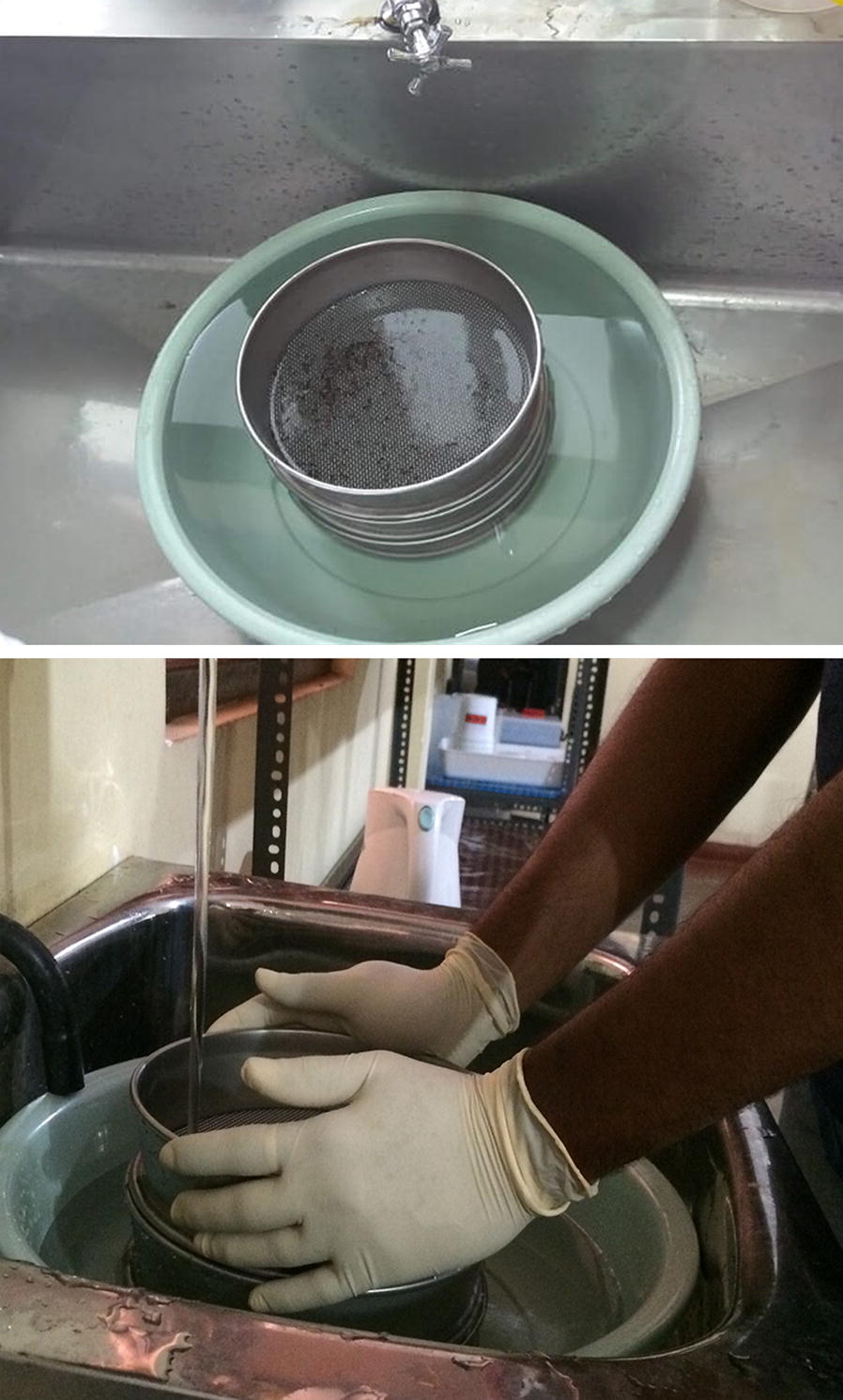
Setup of the standard metal sieving method for pupal separation

A set of Retsch Test Sieves (Glenammer Engineering Ltd, Ayr, Ayrshire) with 1.60, 1.40, 1.25 and 1.12 mm pore sizes were used for the mechanical sex separation. The sieves were precisely positioned over a sluiceway keeping the sieve with the largest pore size on top (Fig. 1). A batch of 300 *Ae. aegypti* pupae (32 h after the onset of pupation) was introduced in to the topmost sieve plate and allowed to pass through by providing a water flow according to the standard operating procedures, following the principle of wet sieving used in ecological monitoring [35].

Separated pupae in each sieve were placed in appropriately-labeled separate plastic cups and were reared to the adult stage. Adult male and female mosquitoes were identified and enumerated after emergence. Dead pupae were identified as male and female under a light microscope according to the differences in the genital lobe shape [36]. The experiment was repeated five times to ensure the accuracy of the findings. The same procedure was followed for *Ae. albopictus*.

#### Fay-Morlan method

Batches of 300 *Ae. aegypti* or *Ae. albopictus* pupae (32 h after onset of pupation) in aquatic culture medium, were introduced into the Fay-Morlan glass plate separator (M5412, John W. Hock Company, Gainesville, USA). The distance between the glass plates was adjusted by using knobs on the apparatus, to yield separate layers of pupae based on their body size. Separated pupae were flushed into separate trays using a mild water flow after loosening the glass plates [37]. The isolated pupae were placed in plastic cups and were reared to the adult stage. Male and female adult *Ae. aegypti* mosquitoes were separated from the collection *via* visual observation using achromatic magnification lenses (×10). Dead pupae were also counted and sorted to gender using the differences in the genital lobe shape (at the end of the pupal abdominal segments just below the paddles) under a light microscope [36]. The experiment was repeated for five times to ensure the accuracy of the findings.

### Behavioural sex separation

#### Spiking blood meals with ivermectin and spinosad at different concentrations

Blood meals (defrosted bovine blood treated with EDTA) were prepared in 50 ml centrifuge tubes containing various concentrations (2, 4, 6, 8 and 10 ppm) of ivermectin [1% w/v injection (BP. Vet), Star Laboratory Pvt Ltd, Lahore, Pakistan] and spinosad (spinosyn A + spinosyn D, 12% w/v preparation; Dow AgroSciences LLC, Indianapolis, USA). The blood was warmed in a water bath and subsequently poured into a Hemotek device (PS-6 System, Discovery Workshops) that maintained the temperature of the blood at 35 ± 1 °C and was placed on the top of 30 × 30 × 30 cm cages containing 300 mosquitoes (1:1 sex ratio) for a period of 2 h for both *Ae. aegypti* and *Ae. albopictus*. Six-day-old adult mosquitoes of both species were used for the experiments. Dead mosquitoes were enumerated at 6 h intervals for 3 days, after the initiation of the experimental trials. Mosquitoes in all cages were provided with 10% sucrose solution *ad libitum*. The experiment was repeated 5 times at each concentration of mosquito toxicants for both *Ae. aegypti* and *Ae. albopictus*. The most effective concentration of toxicants in eliminating females identified in this experiment was selected to be used for the next experiment, described below.

#### Feeding of the most effective toxin concentration initially (0 h) and at 24 h intervals

The 8 ppm concentration, which caused 100% mortality for both species, was selected for further trials. Six-day-old *Ae. aegypti* or *Ae. albopictus* colonies (a batch of 300 mosquitoes with a 1:1 sex ratio) were fed with 8 ppm ivermectin and spinosad separately, initially and at 24 h intervals, with a 2 h feeding duration. The experiment was repeated 5 times for each mosquito species.

#### Mixed feeding of two mosquito toxicants with the same concentration

The experiment was carried out with cattle blood containing a combined mixture of ivermectin and spinosad in a 1:1 ratio at different concentrations (2 ppm: 2 ppm; 4 ppm: 4 ppm; 6 ppm: 6 ppm; 8 ppm: 8 ppm). These mixtures of spiked blood were provided to 6-day-old *Ae. aegypti* or *Ae. albopictus* colonies (with each batch of 300 having a 1:1 sex ratio) initially and then at 24 h intervals with a 2 h feeding duration. The experiment was repeated 5 times.

### Statistical analysis and interpretation of data

Blood-feeding rates of female mosquitoes were calculated for each treatment as the percentage of the number of blood-fed females out of the total number of females in each cage. Mortality rates were calculated for males and females separately as a percentage of the number of dead males or females out of total number of males and females (blood-fed) in each cage. One-way analysis of variance (ANOVA) followed by Tukey’s *post-hoc* pairwise comparison tests were used to investigate the difference in feeding and mortality rates of males and females at different concentrations of ivermectin and spinosad. Probit analysis was performed to determine the toxicities of ivermectin and spinosad, and to calculate the LD_50_, LD_95_ and LD_99_ values for both chemicals separately.

Male and female pupae in each band of the Fay-Morlan glass plate experiment were analyzed using a Chi-square test of independence to evaluate the efficiency of sex separation and also to analyze whether the percentage of male and female pupae separated at different pore sizes (1.12, 1.25, 1.40 and 1.60 mm) differed in the standard sieving method.

## Results

### Standard sieving method

The majority of the male pupae were retained in the 1.12 mm pore sized sieve in both *Ae. aegypti* (73%, *n* = 438) and *Ae. albopictus* (69%, *n* = 483) followed by pore sizes < 1.12 and 1.25 mm. On the other hand, the majority of *Ae. aegypti* (63%, *n* = 441) and *Ae. albopictus* females (66%, *n* = 396) were found at the sieve with a pore size of 1.25 mm followed by 1.12, < 1.12 and 1.40 mm (Table 1). The distribution of sexes captured at different pore sizes differed significantly for both *Ae. aegypti* (*χ*^2^ = 58.38, *df*  = 4, *P* < 0.001) and *Ae. albopictus* (*χ*^2^ = 41.34, *df* = 4, *P* < 0.001).

**Table 1:** Percentages of male and female pupae (mean ± standard error) of *Ae. aegypti* and *Ae. albopictus*, separated at different pore sizes by the standard sieving method

Species (*N* = 1500)	Percentage of male and female pupae retained			
	Pore size, mm	Percentage of retained pupae		Pupal mortality (%)
		Male pupae in sieve (%)	Female pupae in sieve (%)	
*Ae. aegypti* (M: 600; F: 900)	< 1.12	26 ± 2.5 (*n* = 156)	5 ± 1.0 (*n* = 35)	0
	1.12	73 ± 3.5 (*n* = 438)	28 ± 2.5 (*n* = 196)	4 ± 0.5 (*n* = 60)
	1.25	1 ± 0.2 (*n* = 6)	63 ± 3.6 (*n* = 441)	3 ± 0.8 (*n* = 45)
	1.4	0	4 ± 0.8 (*n* = 28)	0
	1.6	0	0	0
*Ae. albopictus* (M: 700; F: 800)	< 1.12	24 ± 2.2 (*n* = 168)	4 ± 0.5 (*n* = 24)	0
	1.12	69 ± 3.5 (*n* = 483)	27 ± 2.6 (*n* = 162)	5 ± 0.4 (*n* = 75)
	1.25	7 ± 1.0 (*n* = 49)	66 ± 4.0 (*n* = 396)	4 ± 0.4 (*n* = 60)
	1.4	0	3 ± 0.4 (*n* = 18)	0
	1.6	0	0	0

*Note:* Values are mean ± SE, with the number of pupae in parenthesis. The male and female proportions in the total sample are stated in the first column

*Abbreviations*: F, female; M, male

### Fay-Morlan glass plate separation method

**Fig. 2:**
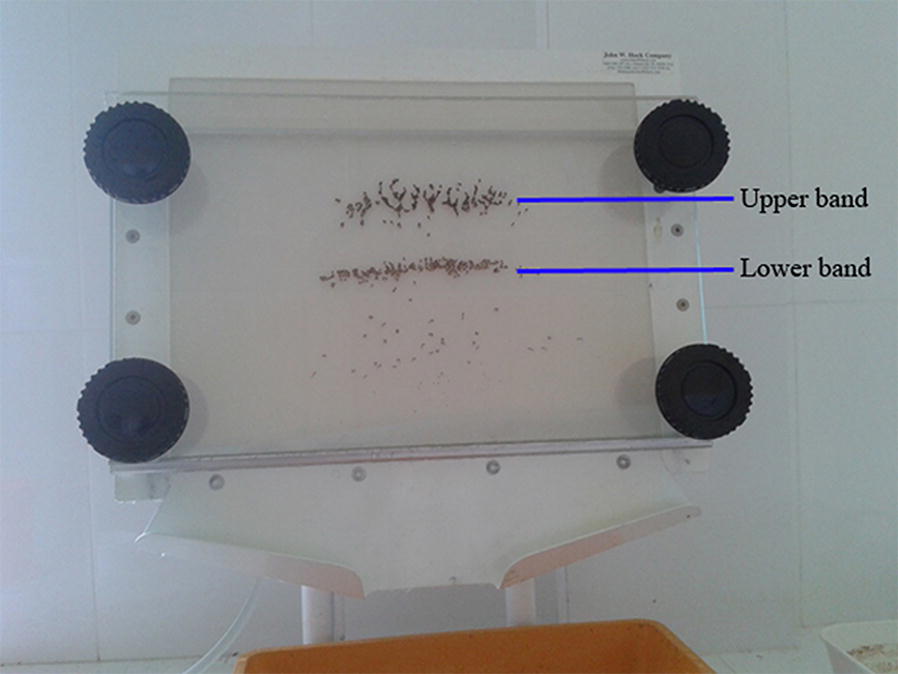
Pupal separation by the Fay-Morlan glass plate separator showing the lower and upper bands

**Fig. 3:**
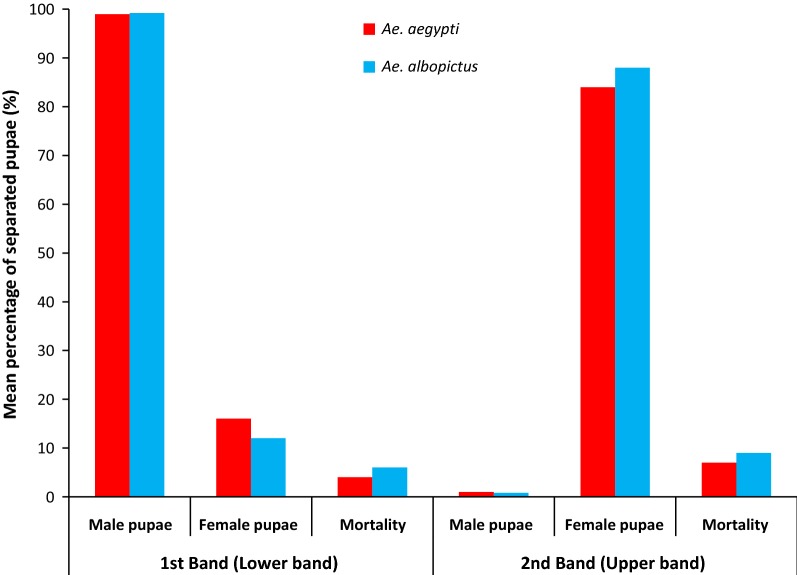
Mean percentages of separated male and female pupae of *Ae. aegypti* and *Ae. albopictus* by the Fay-Morlan glass plate separator

Two separated rows of pupae were observed in the Fay-Morlan glass plate separator as upper and lower bands (Fig. 2). Adult rearing results of the *Ae. aegypti* pupae separated in the lower band indicated that 99.0% (*n* = 643) of male pupae were captured in the lower band with 16.0% (*n* = 136) of females as contamination (Fig. 2). The upper band of the Fay-Morlan glass plate separator captured the remaining 1% of males (*n* = 7) and 84% of females (*n* = 714). The mortality of pupae was 4% and 7% in each band, respectively (Fig. 2). For *Ae. albopictus*, 99.2% (*n* = 714) of male pupae were found in the lower band with 12.0% (*n* = 94) of females. In the upper band, all remaining female (88.0%, *n* = 686) and male (0.8%, *n* = 6) pupae were retained (Fig. 3). Mortality observed was 6% and 9% in the lower and upper bands for *Ae. albopictus*, respectively (Fig. 3). The percentage sex separation of *Ae. aegypti* (*χ*^2^ = 22.57, *df*  = 1, *P* < 0.001) and *Ae. albopictus* (*χ*^2^ = 18.59, *df*  = 1, *P* < 0.001) separated at each band differed significantly.

### Spiking blood meals with different concentrations of ivermectin and spinosad

Feeding rates of both *Ae. aegypti* and *Ae. albopictus* decreased gradually with the increasing concentrations of ivermectin and spinosad (one-way ANOVA: *F*_(5,24)_ = 3.617, *P* = 0.014 and *F*_(5,24)_ = 3.373, *P* = 0.019, respectively) as indicated in Table 2. The female mortality varied among different concentrations of each toxicant for both *Ae. aegypti* (*F*_(5,24)_ = 3.291, *P* = 0.021) and *Ae. albopictus* (*F*_(5,24)_ = 3.001, *P* = 0.030). As indicated in Table 2, the minimum ivermectin concentration delivered within 48 h that achieved 100% female mortality (*n* = 750) for both species was 8 ppm.

**Table 2:** Percentage feeding and cumulative mortality rates (mean ± standard error) of *Ae. aegypti* and *Ae. albopictus* with single feeding of ivermectin and spinosad

Species/toxicant	Concentration (ppm)	Fed females (%)	Percentage mortality (%)					
			24 h		48 h		72 h	
			Male	Female	Male	Female	Male	Female
*Ae. albopictus*								
Ivermectin	0 (Control)	98.0 ± 1.23 (*n* = 735)	0.7 ± 0.54 (*n* = 5)	1.4 ± 0.98 (*n* = 10)	2.0 ± 1.50 (*n* = 15)	5.4 ± 1.89 (*n* = 40)	3.3 ± 1.38 (*n* = 25)	7.5 ± 2.35 (*n* = 55)
	2	98.7 ± 1.46 (*n* = 740)	0.7 ± 0.40 (*n* = 5)	11.5 ± 9.35 (*n* = 85)	2.0 ± 1.50 (*n* = 15)	35 ± 5.36 (*n* = 259)	4.0 ± 2.32 (*n* = 30)	49.3 ± 11.25 (*n* = 364)
	4	100.0 ± 0.2 (*n* = 750)	0 ± 0 (*n* = 0)	36.0 ± 12.35 (*n* = 270)	1.3 ± 0.87 (*n* = 10)	65.3 ± 10.32 (*n* = 490)	5.3 ± 1.52 (*n* = 40)	89.3 ± 9.23 (*n* = 670)
	6	98.7 ± 0.98 (*n* = 740)	0.7 ± 0.54 (*n* = 8)	41.2 ± 8.36 (*n* = 305)	2.7 ± 2.54 (*n* = 20)	87.2 ± 13.26 (*n* = 645)	4.0 ± 1.35 (*n* = 30)	95.9 ± 2.54 (*n* = 709)
	8	94.5 ± 1.54 (*n* = 709)	1.3 ± 0.87 (*n* = 10)	63.9 ± 8.52 (*n* = 453)	3.3 ± 1.23 (*n* = 25)	100.0 ± 2.50 (*n* = 709)	4.7 ± 1.87 (*n* = 35)	100.0 ± 0.00 (*n* = 709)
	10	64.7 ± 1.78 (*n* = 484)	1.3 ± 0.87 (*n* = 10)	91.8 ± 7.56 (*n* = 444)	3.3 ± 1.23 (*n* = 25)	100.0 ± 1.40 (*n* = 484)	5.7 ± 2.35 (*n* = 43)	100.0 ± 0.00 (*n* = 484)
Spinosad	0 (Control)	98.0 ± 0.87 (*n* = 735)	0.7 ± 0.54 (*n* = 5)	1.4 ± 0.84 (*n* = 10)	2.0 ± 1.23 (*n* = 15)	5.4 ± 1.57 (*n* = 40)	3.3 ± 1.85 (*n* = 25)	7.5 ± 2.31 (*n* = 55)
	2	97.3 ± 1.45 (*n* = 730)	2.0 ± 0.4 (*n* = 15)	7.5 ± 0.55 (*n* = 55)	1.3 ± 0.87 (*n* = 10)	43.2 ± 10.23 (*n* = 315)	2.7 ± 0.98 (*n* = 20)	66.4 ± 5.36 (*n* = 64)
	4	98.7 ± 1.95 (*n* = 740)	2.0 ± 1.50 (*n* = 15)	15.5 ± 2.68 (*n* = 113)	2.0 ± 1.50 (*n* = 15)	59.5 ± 12.32 (*n* = 440)	5.3 ± 1.98 (*n* = 40)	84.5 ± 6.37 (*n* = 634)
	6	97.3 ± 0.79 (*n* = 730)	3.0 ± 20 (*n* = 22)	41.8 ± 5.62 (*n* = 305)	2.7 ± 0.85 (*n* = 20)	76.7 ± 15.36 (*n* = 560)	3.3 ± 1.28 (*n* = 25)	95.2 ± 5.21 (*n* = 695)
	8	98.0 ± 1.54 (*n* = 735)	0.7 ± 0.54 (*n* = 5)	53.7 ± 10.68 (*n* = 395)	1.3 ± 0.87 (*n* = 10)	86.4 ± 13.25 (*n* = 635)	2.7 ± 0.97 (*n* = 20)	100.0 ± 0.00 (*n* = 735)
	10	79.4 ± 1.23 (*n* = 595)	2.0 ± 1.50 (*n* = 15)	85.5 ± 9.34 (*n* = 642)	3.3 ± 0.98 (*n* = 25)	100.0 ± 1.00 (*n* = 595)	4.0 ± 2.54 (*n* = 30)	100.0 ± 0.00 (*n* = 595)
*Ae. aegypti*								
Ivermectin	0 (Control)	100.0 ± 0.4 (*n* = 750)	1.3 ± 0.87 (*n* = 10)	2.0 ± 0.76 (*n* = 15)	1.3 ± 0.87 (*n* = 10)	4.7 ± 1.58 (*n* = 35)	3.3 ± 1.26 (*n* = 25)	6.0 ± 2.32 (*n* = 45)
	2	100.0 ± 0.25 (*n* = 750)	0.7 ± 0.54 (*n* = 5)	14.0 ± 5.68 (*n* = 105)	2.0 ± 0.95 (*n* = 15)	42.0 ± 5.36 (*n* = 315)	4.0 ± 1.28 (*n* = 30)	59.3 ± 5.36 (*n* = 445)
	4	98.0 ± 1.45 (*n* = 735)	0.7 ± 0.54 (*n* = 5)	23.1 ± 6.95 (*n* = 170)	1.3 ± 0.83 (*n* = 10)	65.3 ± 9.35 (*n* = 480)	5.3 ± 2.35 (*n* = 40)	91.8 ± 6.34 (*n* = 674)
	6	100.0 ± 0.8 (*n* = 750)	0.7 ± 0.54 (*n* = 5)	30.0 ± 7.36 (*n* = 225)	2.0 ± 0.85 (*n* = 15)	88.0 ± 12.35 (*n* = 660)	4.0 ± 1.78 (*n* = 30)	98.0 ± 2.34 (*n* = 735)
	8	91.7 ± 11.27 (*n* = 686)	0.7 ± 0.54 (*n* = 5)	66.2 ± 5.62 (*n* = 454)	1.3 ± 0.95 (*n* = 10)	100.0 ± 2.40 (*n* = 686)	4.7 ± 1.24 (*n* = 35)	100.0 ± 0.00 (*n* = 686)
	10	50.7 ± 9.31 (*n* = 380)	1.3 ± 0.87 (*n* = 10)	100.0 ± 1.5 (*n* = 380)	3.3 ± 0.97 (*n* = 25)	100.0 ± 2.00 (*n* = 380)	6.0 ± 2.35 (*n* = 45)	100.0 ± 0.00 (*n* = 380)
Spinosad	0 (Control)	99.3 ± 1.34 (*n* = 745)	1.3 ± 0.87 (*n* = 10)	4.2 ± 2.32 (*n* = 31)	1.3 ± 1.23 (*n* = 10)	4.8 ± 1.26 (*n* = 36)	3.3 ± 1.58 (*n* = 25)	6.0 ± 1.68 (*n* = 48)
	2	97.4 ± 0.66 (*n* = 730)	0.7 ± 0.54 (*n* = 5)	15.9 ± 2.67 (*n* = 120)	1.3 ± 1.23 (*n* = 10)	44.9 ± 9.35 (*n* = 328)	4.0 ± 2.31 (*n* = 30)	57.2 ± 9.35 (*n* = 418)
	4	98.0 ± 1.23 (*n* = 735)	0.7 ± 0.54 (*n* = 5)	24.6 ± 5.36 (*n* = 181)	1.3 ± 0.97 (*n* = 10)	62.1 ± 5.39 (*n* = 456)	6.2 ± 1.20 (*n* = 47)	92.3 ± 5.31 (*n* = 678)
	6	97.9 ± 1.08 (*n* = 734)	0 ± 0 (*n* = 0)	26.8 ± 3.68 (*n* = 198)	2.0 ± 0.97 (*n* = 15)	77.4 ± 8.36 (*n* = 76)	4.0 ± 2.35 (*n* = 30)	94.1 ± 8.64 (*n* = 691)
	8	97.3 ± 2.34 (*n* = 729)	0.7 ± 0.54 (*n* = 5)	74.2 ± 5.68 (*n* = 541)	1.3 ± 1.28 (*n* = 10)	100.0 ± 1.0 (*n* = 729)	5.3 ± 3.21 (*n* = 40)	100.0 ± 0.00 (*n* = 729)
	10	54.3 ± 8.35 (*n* = 407)	1.3 ± 0.87 (*n* = 10)	100.0 ± 1.20 (*n* = 407)	3.3 ± 1.23 (*n* = 25)	100.0 ± 0.6 (*n* = 407)	6.0 ± 1.54 (*n* = 45)	100.0 ± 0.00 (*n* = 407)

*Note:* Values are mean ± SE, with the number of pupae in parenthesis. A sample size of 1500 mosquitoes from both species were tested for two toxicants, separately at each concentration. The percentage mortality of females was calculated based on the number of blood-fed females at each concentration

For spinosad, 100% mortality (*n* = 750) was observed at 10 ppm for *Ae. albopictus* within 48 h, whereas 100% (*n* = 750) mortality in *Ae. aegypti* was observed at 8 ppm 48 h after the initial blood meal. Feeding rates of *Ae. aegypti* and *Ae. albopictus* females at 10 ppm were significantly lower than that of 8 ppm for both toxicants (*F*_(5,24)_ = 3.211, *P* = 0.023 and *F*_(5,24)_ = 6.721, *P* < 0.001, respectively). Therefore, the 8 ppm concentration of ivermectin and spinosad could be suggested as the best concentration for sex separation as a single application. The male mortality of *Ae. aegypti* and *Ae. albopictus* in control and experimental settings was not significantly different for either ivermectin or spinosad (*P* > 0.05).

### Probit analysis and lethal dosage (LD) calculations for ivermectin and spinosad

**Fig. 4:**
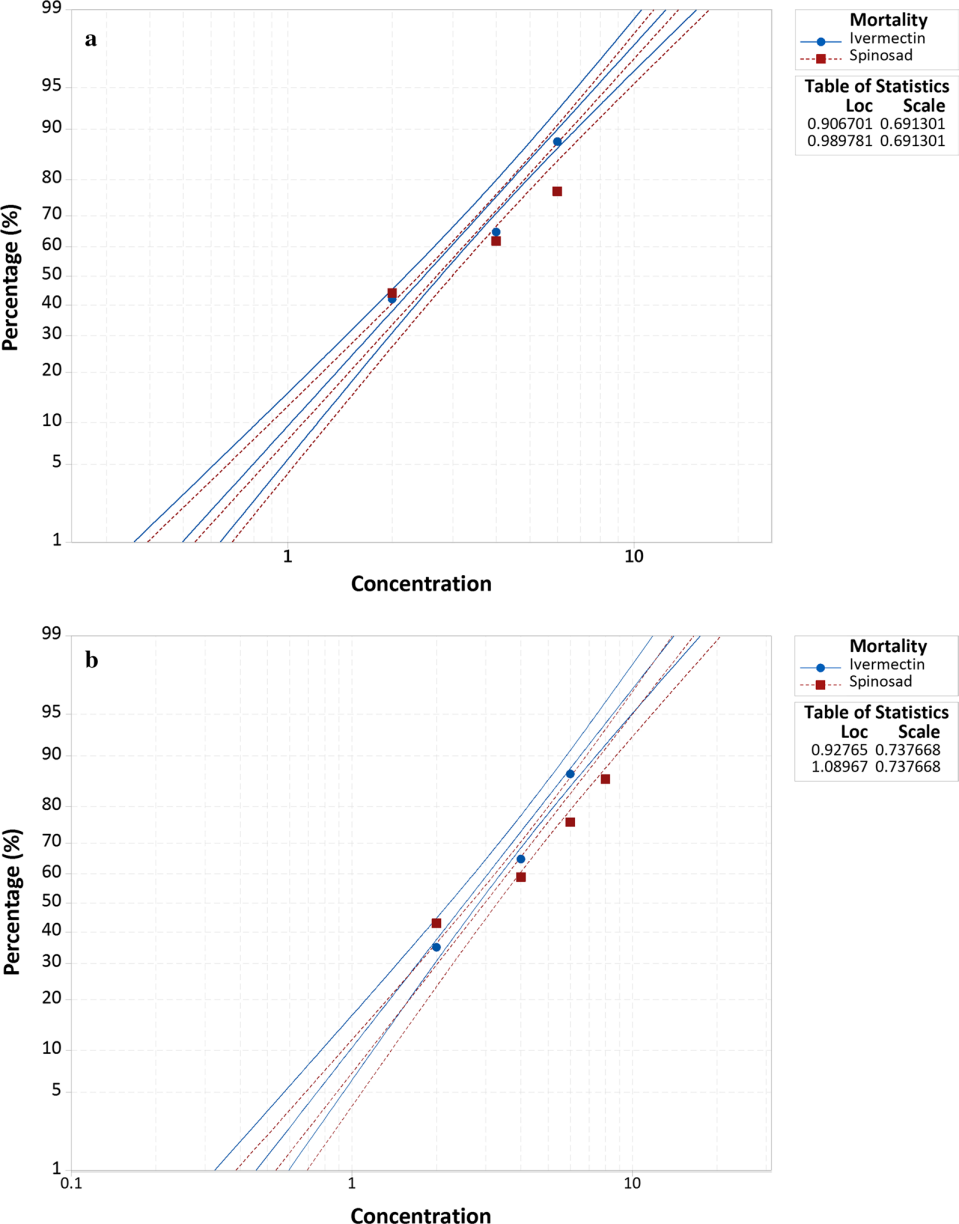
Probit analysis for 24 h mortality of (**a**) *Ae. aegypti* and (**b**) *Ae. albopictus* after providing one spiked blood meal with ivermectin and spinosad

For both *Ae. aegypti* and *Ae. albopictus*, ivermectin produced a relatively steeper slope in the mortality than spinosad within 48 h, as denoted by the Probit analysis (Fig. 4). This suggests that the toxic effect of ivermectin on *Ae. aegypti* and *Ae. albopictus*is is relatively higher than that of spinosad (Fig. 4). Using a single, rather than multiple, feeding approach, the calculated LD values for ivermectin and spinosad from 48 h mortality of each species also showed relatively lower LD values for ivermectin than spinosad (Table 3).

**Table 3:** Lethal dose (LD) values (LD ± standard error, SE) for ivermectin and spinosad at 48 h for *Ae. aegypti* and *Ae. albopictus*

Lethal dose	*Ae. aegypti*		*Ae. albopictus*	
	Spinosad (ppm)	Ivermectin (ppm)	Spinosad (ppm)	Ivermectin (ppm)
LD_50_	2.61 ± 0.18	2.55 ± 0.15	2.72 ± 0.21	2.76 ± 0.14
LD_95_	8.82 ± 0.79	7.36 ± 0.57	11.99 ± 1.43	7.31 ± 0.52
LD_99_	14.59 ± 1.92	11.41 ± 1.27	22.15 ± 3.94	10.95 ± 1.11

*Note:* Values are mean ± SE, calculated from the Probit analysis

### Feeding of the effective toxin concentration (8 ppm) at 24- and 48-h intervals

**Fig. 5:**
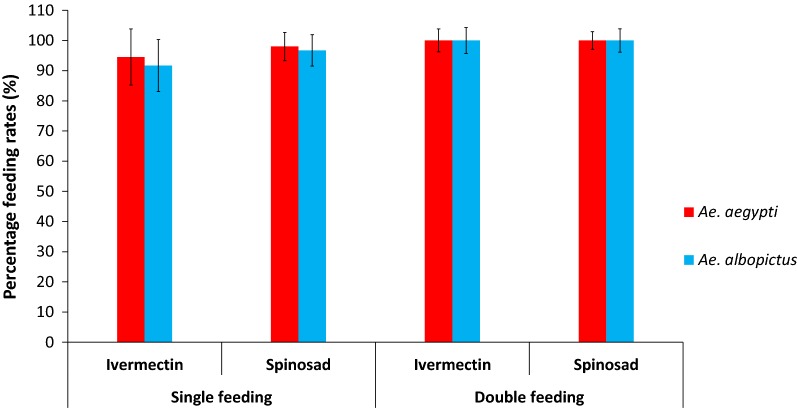
Blood-feeding rates of female *Ae. aegypti* and *Ae. albopictus* for spiked blood with 8 ppm concentration of ivermectin and spinosad, separately initially (single feeding) and after 24 h since the initial blood meal (double feeding)

**Fig. 6:**
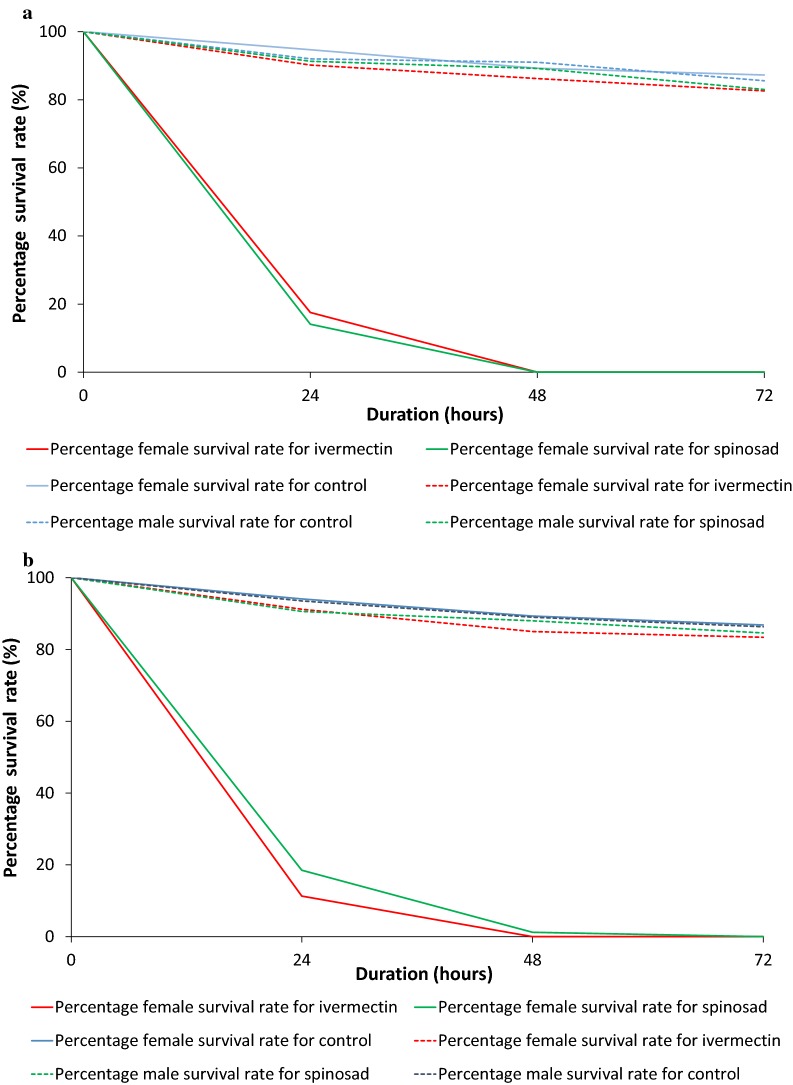
Survival rates of (**a**) *Ae. aegypti* and (**b**) *Ae. albopictus* females using an 8 ppm concentration of ivermectin and spinosad under double feeding

The feeding rates of *Ae. aegypti* and *Ae. albopictus* were less than 100% at the initial feeding; however, at 24 h, all females of both species were engorged with the blood meal. The maximum feeding rate of the females of both species was observed after providing two blood meals initially followed by another at 24 h duration (Fig. 5). Both ivermectin and spinosad showed a 100% elimination of *Ae. aegypti* within 48 h (Fig. 6). *Aedes albopictus* showed 100% mortality within 24–48 h after feeding with ivermectin and spinosad (Fig. 6). Male mortality of *Ae. aegypti* and *Ae. albopictus* at 48 h in the experiment did not differ significantly from controls (*P* > 0.05).

### Mixed feeding of two mosquito toxicants with same concentrations

**Fig. 7:**
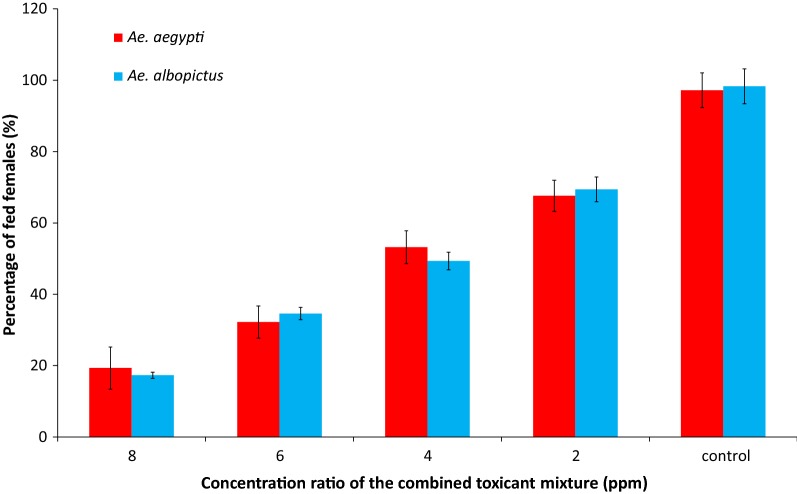
Blood-feeding rates of *Ae. aegypti* and *Ae. albopictus* females with a combined toxicant mixture containing ivermectin and spinosad with equal concentrations

**Fig. 8:**
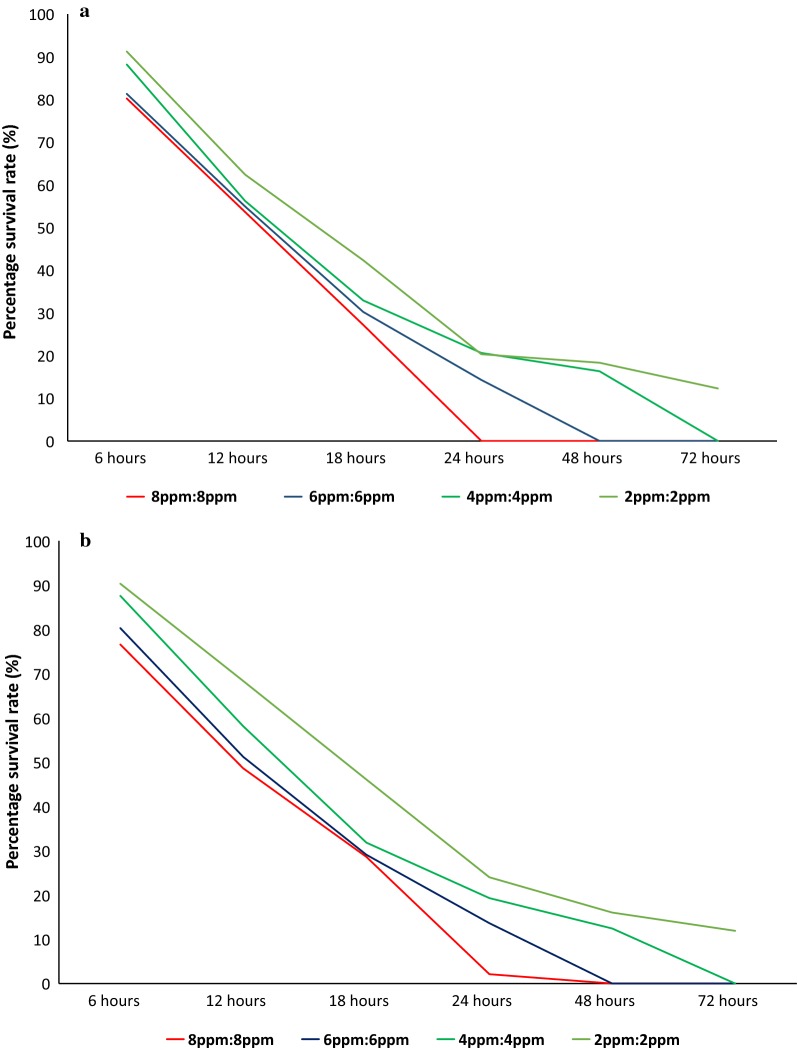
Survival rates of *Ae. aegypti* (**a**) and *Ae. albopictus* (**b**) females using an equal concentration mixture of ivermectin and spinosad after providing two spiked blood meals, an initial meal at 24 h and a second at 48 h

Feeding rates of both *Ae. aegypti* and *Ae. albopictus* showed a negative correlation with the concentrations of both toxicants (Fig. 7). A 100% feeding rate was observed in *Ae. aegypti* in the control treatment, while the blood-feeding rates of mosquitoes varied significantly among different concentration mixtures in both *Ae. aegypti* (one-way ANOVA, *F*_(4,21)_ = 3.112, *P* = 0.037) and *Ae. albopictus* (*F*_(4,21)_ = 3.425, *P* = 0.026). The spiked blood meal containing a 1:1 mixture of ivermectin and spinosad (each at 8 ppm concentration) produced 100% female mortality for *Ae. aegypti* after 24 h of initial feeding (Fig. 8). However, 100% female mortality was achieved in *Ae. albopictus* nearly 40 h after the initial blood meal contaminated with 8 ppm of the toxicant mixture (Fig. 8).

## Discussion

To develop efficient sex separation methods for SIT and IIT programmes, it is essential to evaluate the efficacy and applicability of different approaches such as the use of basic genetic/molecular tools as well as available mechanical and behavioral methods. Genetic sexing strains (GSS) mostly rely on genes conferring insecticide resistance, with the male being heterozygous for resistance due to Y chromosome translocation, while females are homozygous susceptible. However, these strains may have some stability issues [13, 38, 39]. Therefore, more attention has focused on separation of insects through basic mechanical and behavioral methods.

On average, male mosquitoes develop to the pupal stage more rapidly than females, a phenomenon called protandry. This has been exploited for *Aedes* and *Culex* mosquitoes [40] for sex separation, and could also be considered for other species for which methods are not yet available. Most pupae that emerge on the first day are males, with a relatively smaller body size than the females, more of which emerge on the second or third day. Therefore, it is possible to separate sexes at the pupal stage due to the size dimorphism between male and female pupae, since females are generally larger. A number of methods such as (i) sieving using grids of regularly spaced nylon wires [35], (ii) using the glass plate sex separation system [37, 41] and (iii) using the McCray adjustable slit separation system [42], have already exploited the size dimorphism between males and females in sex separation of mosquito pupae. The present study evaluated the sieving and glass plate methods. Among these methods the sex separation efficiencies of the first and second mechanical methods were evaluated due to their easy applicability, low capital and maintenance costs [35, 41, 42].

According to the results of the standard sieving method, a pore size of 1.12 mm was identified as the best out of the five sieve sizes tested to separate male pupae in the laboratory colonies of *Ae. aegypti* and *Ae. albopictus*. Mortality of separated pupae was observed only at 1.12 and 1.25 mm pore sizes. This mortality could occur due to mechanical damage when the pupae pass through the metal pores. However, there were no significant differences observed in the mortality rates of pupae at different sieve pore sizes. It is important to state that this method alone cannot achieve full separation of sexes, since only 73% and 69% male separation occurred at the 1.12 mm pore size for *Ae. aegypti* and *Ae. albopictus*, respectively. Therefore, we concluded that this method cannot be considered as a promising method to be used for sex separation of *Aedes* mosquitoes.

A better separation of sexes was detected from the Fay-Morlan glass plate method compared to the sieving method. Approximately 99% of males were identified in the lower band from this method. Therefore, it is suggested that this glass plate separation method can provide a good separation of sexes for mass rearing colonies. This method is easier and faster than a behavioral sex separation method. However, 16% contamination of females is still not negligible, because this will represent a considerable number of females, if tens of thousands of mosquitoes are released during a mass-release programme. Hence, an additional mechanism to achieve 100% sex separation is essential in SIT and IIT approaches.

Sex separation could be achieved at the adult stage by spiking blood with insecticides (malathion, dieldrin) or other mosquito toxins (ivermectin, spinosad) as a behavioural sex separation approach. A study conducted for *Anopheles arabiensis* females with spiked blood of 7.5 ppm ivermectin and spinosad solutions resulted complete elimination of females within 48 and 72 h, respectively, after the first blood-feeding [43]. Use of toxins that have little or no contact or vapor toxicity, such as ivermectin and spinosad, is effective for such applications [13, 43].

According to the present experiment, a 100% mortality rate for fed *Aedes* females of both species was observed at 8 and 10 ppm for ivermectin and spinosad, respectively, within 48 h of the first blood meal. However, a considerable proportion of female mosquitoes were reluctant to feed on spiked blood contaminated with 10 ppm of ivermectin and spinosad compared to lower concentrations. This could be due to elevation of the vapour concentration with increasing concentrations of toxicants, inducing rejection of the blood meal by the female mosquitoes [13]. Therefore, another blood meal was given 24 h after the first blood meal treated with 8 ppm ivermectin and spinosad, separately, that resulted in 100% female mortality for both toxins.

The present study was conducted in cages with high mosquito densities and regular blood-feeding that caused elevated mortality rates in insectary rearing. However, survival of females may be improved by provision of resting sites and additional sugar sources [43]. It is also important to motivate as many of the females to take a blood meal as quickly as possible. This can be achieved by: removing the sugar source 12 h before offering the blood-meal, using fresh blood, and timing the blood-feeding to preferred times of the day. In some mosquito species, virgin females sometimes do not feed on blood, but later take a blood meal once inseminated. Therefore, in application of this method for mass rearing, the female mortality rate could be increased by prolonging blood-feeding for several hours or feeding multiple times per day [43].

Both ivermectin and spinosad induced low levels of cumulative male mortality rates (< 6%) in this experiment. However, mortality did not differ from controls and some mortality in males was expected due to the damage and stress caused while aspirating relatively small and easily damaged male mosquitoes into test cages. Furthermore, removal of the sugar for a longer period may have increased mortality.

It was observed that when both toxicants are used at a 1:1 concentration mixture, effectiveness was comparatively low compared to individual use of toxicants. Therefore, we recommend ivermectin alone as the more effective preparation for behavioral sex separation than spinosad, since LD values of ivermectin are less than for spinosad for both species. According to a previous study, females of *Culex tritaeniorhynchus* was more susceptible to aqueous sucrose baits with spinosad than ivermectin [32]. A previous study on *Ae. aegypti* indicated that the adult survival after ingestion of ivermectin in a blood meal varied between strains. Interestingly, studies have suggested that ivermectin can inhibit nuclear import thereby inhibiting dengue viral replication, which might increase usefulness of ivermectin over spinosad [44, 45].

Relatively low operational cost, easy applicability, less time, limited labor and space requirements remain the major factors favoring the use of mechanical sex separation methods. Meanwhile, high capital cost (for purchasing of the equipment), relatively low efficiencies of sex separation with high female contamination rates, and a notable degree of male mortality are the major limitations. When the spiked blood meal approach for sexing is considered, elimination of 100% of females (through the provision of two consecutive blood meals, initially and after 24 h) is a major advantage. On the other hand, keeping females until blood-feeding age in a mass-rearing facility will significantly increase the space, labor and costs compared to rearing of only males until adulthood (especially in terms of feeding), which is the main difficulty in the spiked blood meal approach. In addition, a large amount of the toxicant will be required, which could be relatively expensive and obtaining blood to provide far more feeds than required for routine stock maintenance could be difficult due to various practical and ethical issues [46]. Furthermore, use of toxicants might cause undesirable impacts on both the mosquitoes and the technical staff involved in mass rearing, and this would need careful assessment before full-scale application.

In addition, the reliability of the method may not remain constant at 100%, as it is dependent on behavioral attributes, which can never be controlled completely and may change over time or in response to other external factors. However, the present study has yielded promising results by showing a 100% mortality of females fed with spiked blood contaminated with 8 ppm concentration of either of the tested toxins under double feeding method. Therefore, it is suggested that if the spiked blood meal approach is used as the second screening for sexes after applying the Fay-Morlan glass plate method, a 100% separation of sexes might be achieved reliably with reduced costs, since the female contamination by Fay-Morlan mechanical separation processes was only 16%. This contamination could be effectively eliminated through a spiked blood meal with ivermectin, since the present study indicated a 100% feeding rate of females and thereby 100% female elimination. Hence, this study suggests a combined approach of Fay-Morlan glass plate separation method followed by behavioral separation of remaining females be feeding a blood meal spiked with relatively low concentration ivermectin may achieve total elimination of female mosquitoes and production of males alone in mass releases for SIT and IIT applications.

## Conclusions

Neither of the mechanical sex separation methods, standard sieving and the Fay-Morlan glass plate separation, were able to produce 100% sex separation of *Ae. aegypti* and *Ae. albopictus* at the pupal stage. Providing an initial spiked blood meal of ivermectin (8 ppm) along with an additional blood meal (24 h later) was capable of eliminating females of both species within 24 to 48 h. Based on these findings, we suggest that the use of a spiked blood meal approach as a secondary screening after applying mechanical glass plate separation method may be capable of producing 100% separation of sexes.

## Abbreviations

MOH: Medical Officer of Health
PHI: Public Health Inspector
ANOVA: one-way analysis of variance
AW-IVM: area-wide integrated vector management
GSS: genetical sex separation
IAEA: International Atomic Energy Agency
IIT: incompatible insect techniques
LD: lethal dosage
RH: relative humidity
RIDL: release of insects with dominant lethality
SIT: sterile insect technique
WHO: World Health Organization
